# The Illustration of Altered Glucose Dependency in Drug-Resistant Cancer Cells

**DOI:** 10.3390/ijms241813928

**Published:** 2023-09-11

**Authors:** Kausik Bishayee, Seung-Hee Lee, Yong Soo Park

**Affiliations:** Department of Anatomy, College of Medicine, The Catholic University of Korea, Seoul 06591, Republic of Korea; seunghui6310@daum.net

**Keywords:** Warburg pathway, pentose phosphate pathway, OXPHOS, drug resistance, metabolic reprogramming

## Abstract

A chemotherapeutic approach is crucial in malignancy management, which is often challenging due to the development of chemoresistance. Over time, chemo-resistant cancer cells rapidly repopulate and metastasize, increasing the recurrence rate in cancer patients. Targeting these destined cancer cells is more troublesome for clinicians, as they share biology and molecular cross-talks with normal cells. However, the recent insights into the metabolic profiles of chemo-resistant cancer cells surprisingly illustrated the activation of distinct pathways compared with chemo-sensitive or primary cancer cells. These distinct metabolic dynamics are vital and contribute to the shift from chemo-sensitivity to chemo-resistance in cancer. This review will discuss the important metabolic alterations in cancer cells that lead to drug resistance.

## 1. Introduction

**Figure 1:**
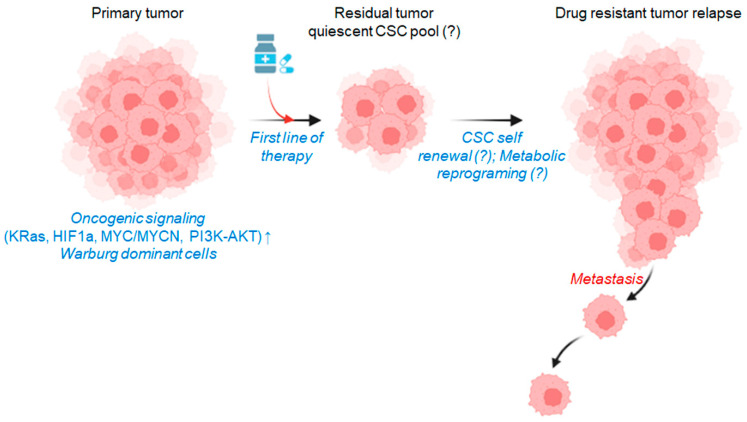
Primary tumors are metabolically dependent on the Warburg pathway for fast energy and carbon sources, and several oncogenic activations feed into it. The first line of cancer therapy with multiple pathway blockers can inhibit the oncogenic activations, but in turn, cancer cells reprogram themselves, and dependency upon the Warburg pathway shifts to other metabolic pathways. Moreover, the theory of CSC’s self-renewal is now a popular area of study, which might be a factor that controls tumor recurrence. The evolved drug-resistant tumors are more aggressive and do metastasize to distant sites in the human body and form secondary tumors. CSC: cancer stem cells, “↑” indicates upregulation and “?” indicates the probable pathway activation (This figure was drawn using the Bio Render app).

Conventional cancer management depends on surgery coupled with chemo- or radiotherapy. In recent times, several kinase pathway blockers are also in use as anticancer drugs [1,2]. Primarily, the conventional cancer therapy process can reduce the tumor mass initially, but unfortunately, repeated treatment of the same anticancer drug does not respond to the cancer cells at a later stage, due to genetic alterations in the cancer cells and tumor microenvironment [3,4]. Thus, the development of drug resistance provides the cancer cells with suitable conditions to repopulate and metastasize at the distal organs [5]. Several recent studies mentioned that therapy resistance against conventional anticancer agents develops due to metabolic reorganizations in the cancer cells [6] (Figure 1).

The ability of cancer cells to modify their metabolism to meet the increased energy demand caused by continuous growth, fast multiplication, and other traits distinctive to neoplastic cells is known as metabolic reprogramming [7]. Metabolic reprogramming is a major obstacle to cancer therapy because metabolite deprivation therapy not only accelerates the growth of tumors but can cause immune cells to become dysfunctional in the tumor microenvironment [8]. Most cancer cells upregulate glucose and/or glutamate uptake to supply carbon for biosynthesis and cope with energy requirements even in the presence of oxygen (Warburg effect). However, targeting this pathway eventually turns on dormant metabolic pathways that can replace the Warburg pathway [9]. For instance, silencing glycolytic genes can restrict the Warburg pathway, but leads to the hyperactivation of the fatty acid oxidation (FAO) pathway and mitochondrial biogenesis in cancer cells [10,11]. Therefore, singly targeting the Warburg pathway may not be enough to supply therapeutic benefits. 

Another popular concept of drug resistance in cancer involves the reawakening of dormant cancer stem cells (CSCs). The dormant CSCs are distinct populations of cancer cells that have traits in common with stem or progenitor cells. Moreover, CSCs do not depend on metabolic pathways that are associated with conventional cancer cell populations; additionally, they hold similar metabolic signatures to noncancerous cells [12,13,14]. Through adaptation to and communication with the tumor microenvironment along with therapeutic pressures, dormant CSCs can activate and grow to form a secondary tumor [15]. More often, the molecular signatures of these secondary tumors vastly differ from the primary ones and are highly therapy-resistant (Figure 1). 

**Figure 2:**
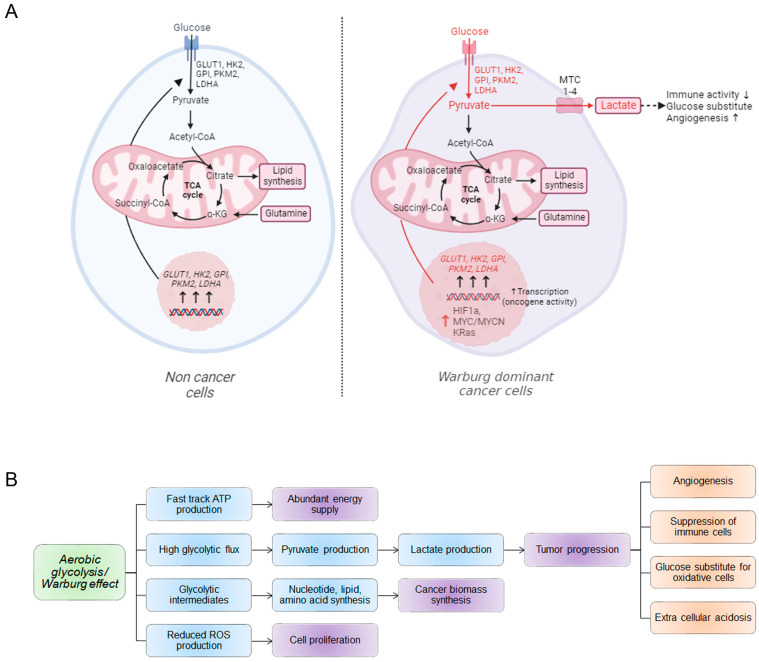
Glucose uptake and its catabolism take place through glycolysis. (**A**) The glycolysis pathway is linked with the tri-carboxylic acid pathway (TCA) for a full breakdown of glucose molecules. The cancer cells upregulate glycolytic enzymes, thus increasing pyruvate and lactate production. Pyruvate acts as an antioxidant, while lactate makes the cellular medium acidic, which helps in cancer growth. HIF-1a, MYC, KRAS, and several other oncogenic proteins are involved in accelerating the transcription of the much-needed glycolytic genes. The glycolysis in cancer cells differs from that in normal cells. In cancer cells, the breakdown of glucose is incomplete, and a large amount of lactate is generated (acronyms: GLUT1: glucose transporter 1, HK2: hexokinase 2, GPI: glucose-6-phosphate isomerase, PKM2: pyruvate kinase M2, LDHA: lactate dehydrogenase A, MTC: monocarboxylate transporters, TCA cycle: tricarboxylic acid cycle, HIF-1α: hypoxia inducible factor 1-alpha, red ↑ indicates upregulation). (This figure was drawn using the Bio Render app). (**B**) The aerobic glycolysis/Warburg pathway is connected with several metabolic processes that sustain the cancer cells’ ability to survive in difficult environments. Other than the catabolic pathway, glycolysis is associated with cancer biomass synthesis and antioxidative mechanisms. The flow describes the association of glycolysis with various mechanisms.

Cells intake glucose from the extracellular space through glucose transporters (GLUT) [16]. Cancer cells upregulate these GLUT families to intake excessive amounts of glucose from the extracellular environment [17,18]. Normal cells use glycolytic pathways to metabolize glucose and produce pyruvate and acetyl-CoA to feed the tricarboxylic acid (TCA) cycle [19]. In cancer cells, the addiction to glucose modifies the glycolytic pathway to produce excessive amounts of lactate and lowers the pyruvate to acetyl-CoA production [20]. The altered glucose metabolism in cancer offers fresh explanations for medication resistance because the molecular mechanisms behind it are still poorly understood (Figure 2A). This article supplies an update on the metabolic reprogramming pathways implicated in tumor resistance.

## 2. Warburg Effect on Cancer Cell Proliferation

Glucose is the main macronutrient used as an energy source for proliferative cancer cells. Therefore, the glucose uptake by the cancer cells exponentially increases and lactate is produced even aerobically in the presence of fully functional mitochondria. This mechanism of the respiration process is known as the Warburg effect, named after Otto Warburg [21,22,23]. The process of aerobic glycolysis is not an optimum process of energy production, which is devoid of mitochondria from their function. Cancer cells adopt this process of glucose metabolism for their need for fast energy sources and other metabolic needs [24,25,26] (Figure 2B). As a result of the Warburg effect, ATP is produced less efficiently for each glucose molecule; however, cancer cells reduce mitochondrial oxidative phosphorylation (OXPHOS), and are thereby able to maintain a supply of carbon-based building blocks otherwise lost as carbon dioxide [27,28]. The Warburg effect is practically linked to the biosynthetic requirements for cancer cell proliferation (Figure 2B). The used glucose serves as the carbon source for the anabolic process required for the proliferation and de novo production of nucleotides for extensive DNA synthesis and lipid metabolism in cancer cells [29,30,31]. 

Elevated glucose metabolism by the Warburg pathway concomitantly increases the lactate production in the tissue microenvironment, making it acidic. Acidosis influences enhanced invasiveness to the cancer cells by altering the tumor–stroma interface [32]. The Warburg pathway hinders the infiltration of immune cells to the tumor soma (Figure 2B). Immune cells require glucose for their activity, the higher glucose uptake capability makes the cancer cells upper-handed in the tumor microenvironment, which limits the availability of glucose for tumor-infiltrating lymphocytes [33]. The Warburg Effect is anticipated to give an overall advantage that promotes a tumor microenvironment favorable to cancer cell growth. Collectively, due to high glycolysis utilization and high lactate levels, cancer cells modulate immune cell infiltration and diminish the effectiveness of immunotherapy [34,35,36]. 

The direct impact of altered glucose metabolism in cancer via signal transduction influences other cellular processes such as the production and regulation of reactive oxygen species (ROS), and the modification of chromatin states [37,38]. The Warburg effect alters mitochondrial redox potential, leading to alterations in the production of ROS [33]. Reduced nicotinamide adenine dinucleotide phosphate (NADPH) is produced via the glycolysis-linked pentose phosphate pathway (PPP). Moreover, NADPH and glutathione, which are produced through de novo serine metabolism, feed into one-carbon metabolism and modulate ROS levels [39,40,41]. These findings establish direct biochemical linkages between aerobic glycolysis and ROS availability, which might alter various signaling systems. The signaling link between the Warburg pathway and histone acetylation can be connected via acetyl-CoA. Acetyl-CoA levels may be sufficient to move cells into the development phase via histone acetylation [42]. When glucose or ATP-citrate lyase is removed, acetylation on numerous histones is reduced, resulting in the decreased transcription of genes involved in glucose metabolism [43,44]. 

It has been shown that the extent to which the Warburg pathway is used by cancer cells can influence their sensitivity to chemotherapeutic agents, and it is known that several oncogenes and mutant tumor suppressors handle the Warburg effect in cancers. Among these, sine oculis homeobox homolog 1 (SIX1), hypoxia-inducible factor 1-alpha (HIF-1α), BHLH transcription factor (MYC), v-MYC avian myelocytomatosis viral oncogene neuroblastoma derived homolog (MYCN), and Kirsten rat sarcoma virus (KRAS) are notable transcription factors [45,46,47,48,49]. 

Oncogenic-driven glycolytic enzymes are switched on to ease the Warburg pathway. The super-activation of the glycolytic enzymes in the glucose metabolism pathway makes it the dominant pathway. Other metabolic pathways, including OXPHOS, go into recessive mode to supplement the proliferation of cancer cells. The fast growth rate of the cancer cells makes them vulnerable to chemotherapy. Studies in established cancer cell lines demonstrated that resistant cells exhibit aerobic glycolysis and increased lactate levels, which are elevated in drug-resistant or metastatic cancers [50]. Thus, the Warburg effect in these cancers may reflect metabolic adaptations related to the emergence of resistance to chemotherapy. Moreover, several glycolytic gene expressions and their activities escalate in chemotherapy-resistant cancer cells. Recently, scientists discovered that lactate could modulate DNA-damage repair processes in resistant cervical carcinoma cells [51].

## 3. Transcriptional Regulation of Warburg Genes

Oncogenic transcription factors are essential for upregulating several genes that favor cancer growth. From that point of view, researchers observed several Warburg genes being transcribed by several oncogenic transcription factors such as the MYC transcription factors, RAS family proteins, HIF-1α, mammalian target of rapamycin (mTOR), neurogenic locus notch homolog protein 1 (Notch1), etc. (Figure 2A) [52].

HIF-1α is the most functional transcription factor in cancer, helping the cells to survive and proliferate in stressful hypoxic conditions. HIF-1α upregulates transcription of all the Warburg genes except for glucose-phosphate isomerase and monocarboxylate transporters (glucose-6-phosphate isomerase (GPI) and SLC16 or SLC5 genes, respectively) [53]. Thus, upregulated HIF-1α always correlates with higher glycolytic flux. Upon phosphorylation, the signal transducer and activator of transcription 3 (STAT3) transcription factor can alternatively activate HIF-1α during oxidative or hypoxic stress [54]. Therefore, STAT3 has a passive role in maintaining the glycolytic flux in cancer cells. Transcriptional cofactors such as peroxisome proliferator-activated receptor gamma coactivator 1-alpha (PGC1α) are upregulated in hepatocarcinoma, glioma, and neuroblastoma. It shows an increase in activity in the hypoxic regions of the tumors. PGC1α upregulates hexokinase 2 (HK2) and GLUT4 in cancer cells [55], but the principal PGC1α targets are mitochondrial biogenesis and OXPHOS genes in cancer cells [56]. 

The MYC proto-oncogene encodes transcription factors c-MYC and MYCN, which bind to the gene promoter’s E-box region to either upregulate or repress transcription. In previous research, we detected that MYCN can accelerate several glycolytic gene transcriptions [10]. A similar instance was observed in glioblastoma with c-MYC. RAS family proteins are also able to manipulate glycolytic genes positively through the RAF/MRK/ERK/c-MYC and PI3K/AKT pathways [57]. A mutation in KRAS (G12V) may induce HIF-1α stabilization. Similarly, c-MYC stabilizes HIF-1α at a normoxic state to alleviate glycolysis [58].

## 4. Activation of Pentose Phosphate Pathway in Warburg Dominant Cancer Cells

The Warburg metabolism is distinguished by high glucose consumption and an incomplete breakdown of lactic acid. With the Warburg pathway, the PPP (also known as the hexose monophosphate shunt) serves many crucial roles in maintaining cancer cell homeostasis. The PPP branched from glycolysis at the first step of glucose-6-phosphate [59]. The PPP is an integral part of the Warburg pathway for benefiting cancer cells and contributes to cancer cells by operating in many ways, such as (I) to inhibit apoptosis by using the reduced form of nicotinamide adenine dinucleotide phosphate (NADPH) [60], (II) as an alternative for energy maintenance via intermediates of the glycolytic products [27], (III) by expanding the cellular pool of nucleic acid bases with genetic material [61], (IV) via boosting glycolysis and therefore lactic acid generation to promote cancer cell survival [62], and (V) by boosting cellular proliferation through nucleic acid, fatty acid, and amino acid biosynthesis [60]. Reduced mitochondria-mediated metabolism with an increase in glucose uptake leads to PPP activation in cancer cells. Activation of PPP is crucial for the cancer cells in maintaining the NDPH/NADP+ ratio, which determines the redox state of the cells by removing ROS elements and preventing cellular death [62]. For instance, the histone-lysine N-methyl transferase 2 (NSD2)-driven tamoxifen-resistant cancers exhibit enriched PPP that elevates NADPH production and reduces ROS levels [63]. Moreover, PPP produces ribose-5-phosphate, which is important for nucleic acid synthesis [64]. According to studies, cancer cells can directly or indirectly change PPP flux to enhance cell survival and proliferation. In hepatocellular carcinoma, breast cancer, and lung cancer, multiple enzymes’ activity from PPP is elevated, and that refers to the poor survival probability [65]. For example, silencing the PPP enzyme glucose-6-phosphate dehydrogenase (G6PD) reduces cancer cell proliferation and migration by inhibiting STAT3 and epithelial-to-mesenchymal transition (EMT) formation [62]. Further, BCL-2 (B-cell lymphoma 2)-associated athanogene (BAG1) can interact with and inhibit G6PD activity, reducing proliferation and DNA synthesis in hepatocellular carcinomas [66]. G6PD inhibition in breast cancer cells increases glycolytic flux and glutamine uptake but reduces lipid biosynthesis [62,67]. G6PD silencing in lung cancer can reduce cell migration and affect the enzymatic activity of multiple PPP elements [68]. 

## 5. Flux Controlling between Warburg and Pentose Phosphate Pathway

HK and phosphofructokinase 1 (PFK1) are two rate-limiting enzymes in the glycolysis pathway. The pentose phosphate pathway (PPP) and glycolysis are the two ways in which the whole flux of glucose is diverted. The rate at which glucose enters glycolysis is regulated by PFK1 [69]. In cancer cells, 6-phosphofructo-2-kinase/fructose-2,6-biphosphatase (PFKFB) controls the cytoplasmic levels of fructose-2,6-bisphosphate and activates PFK1. PFKFBs are often upregulated in pancreatic, colon, prostate, and breast cancers [70]. Protein arginine methyltransferase 1 modifies PFKFB3 to become asymmetrically di-methylated at R131 and R134. Reduced methylation of PFKFB3 suppresses fructose 2,6-bisphosphate in cancer cells, diverting glucose utilization away from glycolysis and toward the pentose phosphate route [71]. The post-translational modifications of the PFKFB isoform, PFKFB3, enable glucose metabolism in response to the stress condition. Further, PFKFBs are widely involved in cell cycle regulation, autophagy, and transcriptional regulation in a non-glycolysis-dependent manner [72]. The fructose-6-phosphate and glyceraldehyde-3-phosphate made in PPP can produce pyruvate by entering the glycolytic pathway [73]. NADPH and ATP are simultaneously produced in this flux mode, and mitochondria can oxidize pyruvate to produce more ATP. Depending on their metabolic needs, cancer cells alter the PPP and glycolysis pathways [64]. 

The metabolic flux diverts to PPP while an increase in ROS is observed. Higher ROS accumulation shuts down GAPDH activity, and the reversibility of the glucose-6-phosphate isomerase increases PPP flux [74,75]. Prolonged inhibition of GAPDH inhibits lactic acid production and acidosis; however, those cells remain glucose-dependent and shift to lipid metabolism. Altered metabolic flux through PPP implies fatty acid synthesis and NADPH production [76,77,78]. It is noteworthy that blocking glycolytic flux rewrites the metabolic signature of the cancer cells, and glucose compensates for the metabolic loss by increasing fatty acid synthesis (FAS) through PPP [79,80,81]. It should be highlighted that cancer cells have an unusual lipid metabolism, and FAS overactivation represents a distinguishing characteristic. FAS is dependent on the energy of reducing equivalents such as NADPH, which is mostly generated in PPP.

## 6. Metabolic Profiling of the Warburg Dominant Cells

Most cancer cells exhibit the Warburg effect, which is characterized by lactate fermentation and excessive glucose absorption. The Warburg effect also causes a metabolic rewiring that increases glutamine consumption and lipid production, both considered cancer hallmarks [82].

The less-celebrated carbon source for cancer cells is glutamine. Many researchers have emphasized the necessity of glutamine as a source of reduced nitrogen that maintains nucleotide biosynthesis and non-essential amino acids [39]. Glutamine is taken up by cells and may be utilized as an amino acid for protein synthesis; however, glutaminase converts it largely to glutamate. Following that, glutamate is transformed into α-ketoglutarate [83]. Glutamine can also be metabolized to pyruvate and ultimately to lactate via malate in a process known as glutaminolysis.

**Figure 3:**
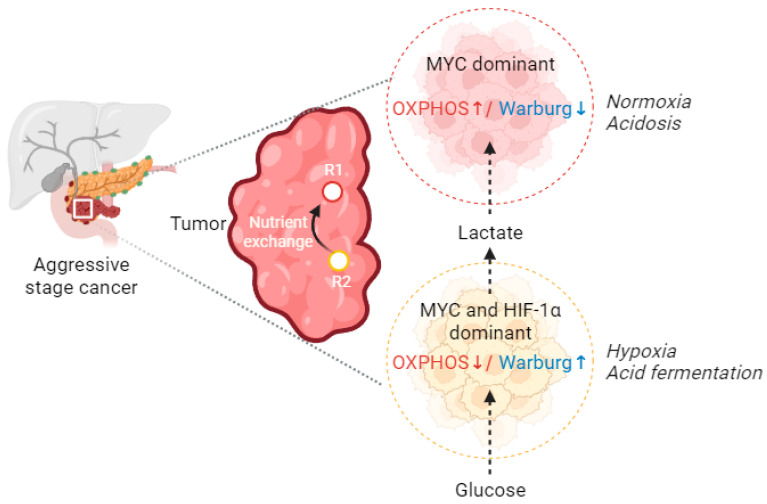
Commensal relationships between tumor cells. The hypoxic tumor cells release lactate, which is recycled and utilized as pyruvate by the more oxygenated cells in OXPHOS. A single oncogene, MYC, can induce aerobic glycolysis, OXPHOS, or mitochondrial biogenesis. Alternatively, when cancer cells are far from an oxygen supply, MYC, in collaboration with HIF-1α, can decrease mitochondrial respiration while not interfering with the mitochondrial biogenesis process (acronyms: OXPHOS: oxidative phosphorylation, R1: tumor region 1, R2: tumor region 2, HIF-1α: hypoxia inducible factor 1-alpha, MYC: BHLH transcription factor). (This figure was drawn using the Bio Render app).

In the tumor microenvironment, the commensal relationships between tumor and stroma need to be noted. The hypoxic tumor cells release lactate, and the more oxygenated stromal cells recycle and are used as pyruvate for OXPHOS [84,85]. Aerobic glycolysis and OXPHOS, or mitochondrial biogenesis, can be driven by a single oncogene, MYC, while cells are located in the vicinity of blood vessels. Alternatively, while cancer cells are distal from the oxygen source, MYC, in cooperation with HIF-1α can suppress mitochondrial respiration without affecting the mitochondrial biogenesis process [86,87] (Figure 3). In the long run, MYC’s ability to promote mitochondrial biogenesis while inhibiting mitochondrial respiration in proliferating cells is not unexpected. Because mitochondria are not only responsible for efficient ATP production in the presence of oxygen, but also produce many other building blocks of a growing cell, these include pyrimidines, the carbon backbone for amino acids, whose synthesis is closely connected to the electron transport chain (ETC) via the enzyme activity of dihydro-orotate dehydrogenase, and citrate, which is extruded into the cytoplasm and transformed to acetyl-CoA for lipid biosynthesis [88].

## 7. Reversing the Warburg Pathway

**Figure 4:**
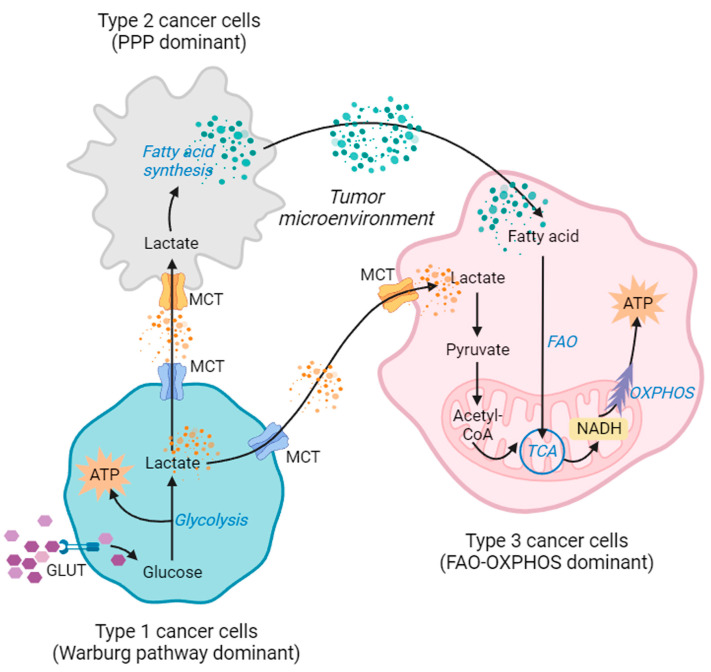
The dynamic microenvironment metabolism is used for symbiotic metabolic reprogramming in tumors. Cancer’s metabolic dependence can be reprogrammed by environmental stress. This suggests that cancer cells may employ a variety of catabolic metabolites to generate energy (acronyms: GLUT: glucose transporters, MTC: monocarboxylate transporters, TCA cycle: tricarboxylic acid cycle, FAO: fatty acid oxidation, ATP: adenosine triphosphate, NADH: reduced nicotinamide adenine dinucleotide, PPP: pentose phosphate pathway, OXPHOS: oxidative phosphorylation). (This figure was drawn using the Bio Render app).

The Warburg effect is crucial to cancer. A way to alleviate the Warburg effect is to convert pyruvate to acetyl-CoA, which could reduce the activation of both the PPP and the glutamate pathway. On the other hand, mitochondrial biogenesis should be elevated to generate ROS. Reversing the Warburg pathway can activate alternative pathway genes, such as the fatty acid oxidation and glutamate pathways [89]. These pathways can supplement acetyl CoA and alpha-ketoglutarate (α-KG) in the TCA cycle. Moreover, they can produce nucleotides and evade ROS-mediated cellular damage (Figure 4).

To avoid the Warburg effect, scientists need to correct two intertwined phenomena. The first is the metabolic bottleneck. The overexpressed pyruvate dehydrogenase kinase (PDK) is the metabolic conjunction of the Warburg pathway, which makes it immortal in cancer metabolism. The Warburg pathway is suppressed by PDK inhibition because it increases the oxidation of glucose carbons in the TCA cycle at the expense of lactate fermentation [90]. The PDK negatively regulates pyruvate dehydrogenase (PDH) [91]. It has been proven that genetically suppressing PDKs slows the growth of cancer cells in tumors and cultures. Restoring PDH activity is essential for the conversion of pyruvate to acetyl-CoA [92]. In cancer cells, PDK is upregulated and converts pyruvate to lactic acid [93]. Suppressing PDK can deliberately increase acetyl-CoA flux into the TCA cycle with simultaneous ATP production. The most well-known inhibitor for PDK is dichloroacetate (DCA), which binds to the pyruvate binding pocket of the PDK enzyme, leading to a change in the conformation. Because of its capacity to activate PDC activity and improve oxidative elimination of lactate, DCA is the most powerful lactate-lowering medication in clinical usage [94]. Because of the role of lactate in tumor immunity, growth, and metastasis, as well as the well-established inverse clinical association of lactate with survival, the ability of DCA to lower tissue and circulating levels of lactate may be an underappreciated, but potent, antitumor action of this and other PDK inhibitors [95]. Earlier reports showed that adding supplements of α-lipoic acid (ALA) to the cancer cells could enhance the activity of PDH. ALA acts as a cofactor for PDH; adding ALA to the cancer cells’ cultures reduces the growth of breast, ovarian, colorectal, and lung cancer cells [96]. Therefore, it is logical to inhibit PDK activity, which would partially restore PDH activity, thereby increasing the flux of pyruvate through the TCA cycle while also inhibiting the production of lactic acid and, most significantly, reducing the flux in the pentose pathway shunt.

The second is the breakdown or elimination of mitochondria. The mitochondria are damaged or destroyed by cytotoxic medicines. When treatment fails, there is a dramatic increase in glucose absorption, as revealed on a positron emission tomography (PET) scan. Chemotherapy resistance has been linked to mitochondrial activity, OXPHOS [97,98]. Mitochondrial biogenesis is regulated by several factors in cancer cells, such as metabolic status, tumor heterogeneity, tissue type, microenvironment, mitophagy, and tumor stage. The most important mitochondrial biogenesis gene is PGC1α, which can be regulated by several factors, as the mammalian target of rapamycin complex 1 (mTORC1) can stimulate and c-MYC negatively regulates PGC1α [11]. PGC-1α-dependent mitochondrial biogenesis may contribute to tumor metastatic potential [99]. The transcriptional networks governing mitochondrial biogenesis affect treatment outcomes by providing cancer cells with the metabolic flexibility to respond to targeted medicines and tumor microenvironments. Resistance to mitogen-activated protein kinase kinase (MEK) inhibitors in V-RAF murine sarcoma viral oncogene homolog B (B-RAF) or neuroblastoma RAS viral (V-Ras) oncogene homolog (N-RAS) mutant melanomas was mediated by PGC-1α overexpression and could be reversed by mTORC1/2 inhibition [100].

## 8. Metabolic Plasticity, a Switch between Warburg and Other Metabolic Pathways

Cancer cells, in contradiction to the Warburg theory, often retain functioning mitochondria [101]. Mitochondria in cancer cells are essential for cell development and survival. The tumor microenvironment promotes energy reprogramming via metabolic pathways such OXPHOS, FAO, and glutaminolysis. This allows cancer cells to gain metabolic plasticity, which is responsible for cancer cells shifting from chemo-sensitive to chemo-resistant. It has been noted that the Warburg effect does not account for the energy production in cancer. It has been proposed that metabolic symbiosis between cancer cells and stromal cells allows cancer cells to produce ATP [102,103]. According to the “Reverse Warburg Effect” model, stromal cells such as cancer-associated fibroblasts, adipocytes, and macrophages produce and transfer metabolites to the cancer cells, where they are used for mitochondrial function and OXPHOS [104,105] (Figure 4).

### 8.1. Warburg–Oxphos Switch

As we mentioned earlier, lactate produced by hypoxic cancer cells contributes to pyruvate synthesis, where the oxidative pathway remains dominant. This alternate metabolic behavior sustains the cancer cells by communicating with each other and producing a symbiotic niche in the tumor microenvironment, which is a profitable utilization and re-utilization of the available substrate in a nutrient-constrained environment [106]. The Warburg mechanism challenges mitochondrial activity—OXPHOS and cells divert the glucose catabolic pathway into several aspects. However, the concept of metabolic plasticity changes the scenario of Warburg dependency in several cancer cells, where OXPHOS remains dominant. Aerobic glycolysis in cancer cells technically supports neighboring tumor cells and permits the delivery of substrates to increase ATP generation, growth, and proliferation via the OXPHOS pathway [27]. This process highlights the relevance of interaction and molecular communication in cancer cell metabolism and shows that the elevation of aerobic glycolysis is not an absolute standard in the tumor microenvironment. The glucose-deprived cancer cells can sustain their metabolic plasticity through the “reversed Warburg effect”, where the extracellular lactate is used up by the cells where glucose deficiency occurs. Therefore, the lactate formed by the Warburg dominant cells acts as the substrate for pyruvate production and is transformed to acetyl-CoA to feed the TCA cycle [104,105].

Genetically, disruption of the Warburg pathway interrupts glycolytic metabolic flux and reroutes the metabolism towards the OXPHOS pathway with little impact on cancer cell growth. Therefore, what we understand is that (1) the constitutive active Warburg pathway does not imply a loss in oxidative metabolism, and (2) mitochondria are capable of taking the lead in adopting cancerous metabolism. Cancer cells depend significantly on cytosolic aerobic glycolysis as well as mitochondrial respiration. 

It has been clear that, in addition to the Warburg pathway, OXPHOS plays a critical role in cancer progression [107]. Research on triple negative breast cancer exhibits both enhanced OXPHOS and a high Warburg effect, with higher mitochondrial respiration and biogenesis activities [108]. It has been shown that HIF-1α, AMPK, and ROS are involved in preserving the oxidative state of the cancer cells while the Warburg pathway is running in the background [109]. Moreover, ROS-mediated activation of RAS, MYC, and cellular sarcoma proto-oncogene tyrosine-protein kinase (c-SRC) restores the mitochondrial biogenesis process [110,111]. This hybrid phenotype enables cancer cells to adapt to different microenvironments to promote tumorigenesis and metastasis, and the simultaneous activities of oncogenes sustain the metabolic plasticity and shifts in cancer cells that incur drug resistance. 

### 8.2. Fatty Acid Synthesis and Oxidation

It should be noted that cancer cells have an unusual lipid metabolism, with hyper-fatty acid synthesis constituting a distinguishing characteristic. Moreover, the cancer cells can lose dependence on the Warburg pathway but remain dependent on glucose. Difficult situations, including therapeutic stress, can inhibit the glycolytic flux in cancer cells. For example, prolonged treatment with the GAPDH inhibitor koningic acid showed a reduction in glycolytic flux in BT-549 but remained dependent on glucose as a carbon source. The BT-549 cells alter the metabolic dependency toward fatty acid synthesis and oxidation pathways [112].

Various cancer cells endogenously synthesize fatty acids for their proliferation and survival; for instance, breast cancer cells synthesize 95% of their fatty acids through the de novo lipogenesis process [113,114]. FAS-related enzyme expressions and activities are upregulated in several cancers [115]. Aerobic glycolysis and PPP directly support the de novo lipid biosynthesis process through the production of acetyl-CoA and NADPH, respectively [27,116]. In PPP, glucose-6-phosphate diverts glucose to generate NADPH and pentose surges. A large amount of NADPH is used during the FAS process. With P53-associated TIGAR, there was a potential blockage against the glucose-6-phosphate enzyme to limit PPP flux and the NADPH synthesis process [117,118]. Alternatively, PKM2 regulates the conversion of phosphoenolpyruvate (PEP) to pyruvate. PKM2 enhances PPP by blocking glycolysis and decreasing metabolite transit through glycolysis, to create additional NADPH [119,120]. PKM2 knockdown in human cancer cell lines and PKM1 replacement reduced tumor development in nude mouse xenografts, which was connected with decreased lactate generation and increased oxygen consumption [121]. Likewise, pyruvate generated from glucose enters the mitochondria and is decarboxylated to acetyl-CoA by pyruvate dehydrogenase (PDH). Through the TCA cycle [122], oxaloacetate (OAA) in mitochondria uses glucose-derived acetyl-CoA to form citrate [123,124]. A portion of the citrate produced by the TCA cycle leaves the mitochondria and is converted by ATP citrate lyase (ACL) to cytosolic acetyl-CoA, which serves as a precursor for fatty acid biosynthesis.

According to the Warburg effect, glycolysis is the source of ATP in tumors, yet ATP levels do not change between cancer cells cultured in the presence and absence of glucose. Several ideas, including metabolic reprograming in the tumor microenvironment, have been presented to explain ATP supply in cancer. However, both hypotheses are dependent on the TCA–OXPHOS route for producing ATP, which contradicts the Warburg effect. Previous findings showed that inhibiting FAO in the presence of glucose significantly reduced ATP synthesis in cancer cells. This confirms that the cancer cells rely on fatty acids to produce ATP via FAO rather than glycolysis [81].

The concept of FAO reprogramming in cancer energy metabolism can be explained by the symbiosis model and the reversed Warburg model (Figure 4), where the lactate is taken up and reused to synthesize fatty acids. The fatty acids are transported to the cancer cells to be utilized in energy production. It has been noted that various fatty acid transporters are often overexpressed in cancer cells [125]. Moreover, increased FAO in the cancer cells could be linked with various mutations and overexpression of the KRAS and MYC oncogenes, respectively [126,127]. Targeting FAO markedly reduces the oxygen consumption rate (OCR) and ATP production. Moreover, as our previous study showed, inhibition of the glycolytic pathway through oncogenic silencing of MYCN reduces glycolytic parameters, but in turn, the FAO pathway becomes dominant for energy reprogramming in the neuroblastoma cell lines. Dual inhibition of the Warburg and FAO pathways (by inhibiting PPARD) reduces cancer cell growth significantly [10]. Thus, metabolic reprogramming gives the cancer cells extra space to maintain energy homeostasis for survival and proliferation. This shows that inhibiting FAO may be a possible treatment method for cancer. Because FAO inhibition limits cancer-dependent catabolism, targeting FAO with anti-proliferation therapies is predicted to have a synergistic therapeutic impact. Because of the Warburg effect, cancer cells do not employ the TCA cycle with carbohydrates. As a result, cancer cells must rely on ETC-OXPHOS to generate ATP from fatty acids.

### 8.3. Glutaminolysis

Other than glucose, glutamine is an alternative energy source and nitrogen donor in cancer cells. It is noted that ample glutamine presence extracellularly promotes cancer cell proliferation and growth. The glutaminolysis is the process of the conversion of glutamine into the intermediates of the TCA cycle and that enables the energy production through NADH [39,128]. Once in the TCA cycle, glutamine carbon skeletons contribute to a hybrid TCA cycle that contains carbons from glucose as well as from glutamine. HIF-1α stimulates pyruvate dehydrogenase kinase (PDK1), which inhibits pyruvate dehydrogenase and the conversion of pyruvate to acetyl-CoA, shunting pyruvate to lactate. Hypoxia, which diverts glucose to lactate, has little effect on glutamine catabolism via the TCA cycle in proliferating cells [129]. Indeed, glutamine may contribute to citrate and lipid metabolism via TCA cycle reversal or reductive carboxylation of α-KG by isocitrate dehydrogenase (IDH) to generate citrate, or by glutamine carbon forward cycling [130,131,132]. Under glucose constraint, the TCA cycle might be reprogrammed and driven purely by glutamine, resulting in citrate composed entirely of glutamine carbons. It is worth noting that certain cells may also take up free fatty acids from media to meet their macromolecular demands, whether for FAO or direct insertion into the membranes of developing cells [133]. 

**Figure 5:**
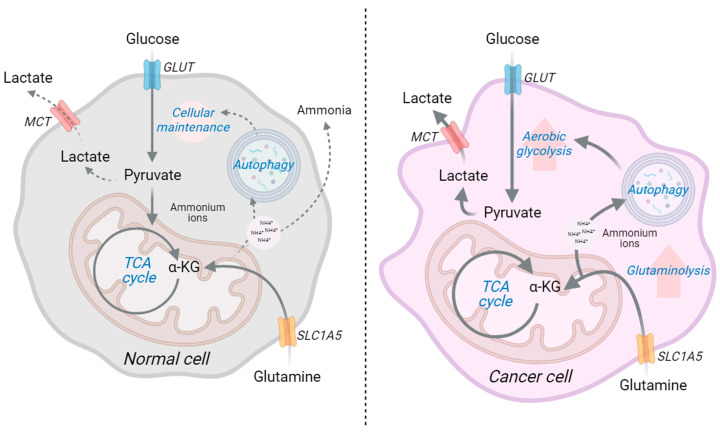
Aerobic glycolysis is a defining feature of cancer metabolism. The majority of the glucose-derived pyruvate is released extracellularly as lactate during this process, and glutamine becomes a conditionally necessary amino acid. Glutaminolysis maintains mitochondrial activity by delivering TCA cycle metabolites such as α-KG and producing a variety of biomolecules such as NEAAs, NADPH, and nucleotides. Increased glutamine flow into the mitochondrial matrix causes metabolic reprogramming toward increased aerobic glycolysis (acronyms: GLUT: glucose transporters, MTC: monocarboxylate transporters, TCA cycle: tricarboxylic acid cycle, α-KG: alpha ketoglutarate, SLC1A5: solute carrier family 1 member 5). (This figure was drawn using the Bio Render app).

Ammonium ions are produced during glutaminolysis by a deamidation process mediated by glutaminase and glutamate dehydrogenase. The majority of ammonium ions are utilized as a nitrogen source for nucleotide production and are eliminated by the urea cycle; nevertheless, an excess of ammonium ions stimulates autophagy. Increased autophagy promotes drug resistance by increasing aerobic glycolysis or the Warburg effect and is important in cancer cell survival, progression, and metastasis [39] (Figure 5). It has been noted that cancer cells that are driven by the MYC and KRAS require glutamine for their survival. MYC can facilitate the glutamine transporter SCL1A5 and GLS enzyme for glutamine uptake and glutaminolysis, respectively [134,135]. Tumorigenic PIK3CA promotes cancer cell glutamine reliance via overexpressing mitochondrial GPT2 activity in colorectal cancer [136]. GLS upregulation has been detected in several tumors, and these enzymes have been discovered to play a role in the metabolic reprogramming of glutamine addiction in cancer. GLS inhibitor, CB-839, is the only one to enter clinical trials; nevertheless, its selectivity for GLS1 and inability to suppress the compensating effect of GLS2 necessitate more investigation [137]. Despite the fact that medicines targeting glutaminolysis have been discovered, none have yet been employed in advanced clinical trials.

## 9. Glucose Metabolism-Based Drug Development

**Figure 6:**
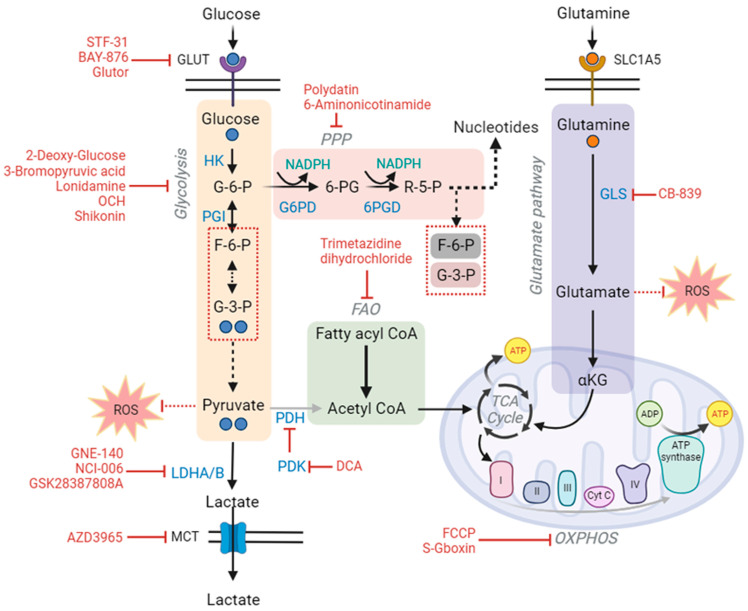
Cancer cells utilize glucose through aerobic glycolysis and the pentose phosphate pathway; both pathways are interlinked and dominant in cancer cells. Aerobic glycolysis is meant to produce fast energy, and the pentose phosphate pathway is used for the biosynthesis of nucleotides and lipids. NADPH and pyruvate can act as antioxidants to regulate ROS generation and evade apoptosis in cancer cells. Inhibition of glycolysis reduces acetyl-CoA production, which could be compensated for by the fatty acid oxidation pathway. Alternatively, inhibition of glycolysis activates the compensatory glutamate pathway. The glutamate pathway has an antioxidative role and can supplement α-ketoglutarate into the TCA cycle. Cancer cells modify metabolic pathways to fulfill their metabolic needs. The alternative pathway activations sustain metabolic homeostasis, which helps in developing drug resistance. Therefore, targeting multiple metabolic pathways is attracting attention for cancer therapy. The pathway inhibitors are indicated in red and enzymes are in blue letters (acronyms: GLUT: glucose transporters, MTC: monocarboxylate transporters, TCA cycle: tricarboxylic acid cycle, α-KG: alpha ketoglutarate, SLC1A5: solute carrier family 1 member 5, HK: hexokinase, PGI: phosphor glucose isomerase/phosphor glucoisomerase, LDHA/B: lactate dehydrogenase A/B, PDH: pyruvate dehydrogenase, PDK: pyruvate dehydrogenase kinase, PPP: pentose phosphate pathway, FAO: fatty acid oxidation, G6PD: glucose 6-phosphate dehydrogenase, F-6-P: fructose-6-phosphate, R-5-P: ribose 5-phosphate, G-3-P: glyceraldehyde 3-phosphate, 6PGD: 6-phosphogluconate dehydrogenase, 6-PG: 6-phosphogluconate dehydrogenase, GLS: glutaminase, FCCP: carbonyl cyanide p-(tri-fluromethoxy) phenyl-hydrazone, OXPHOS: oxidative phosphorylation, ROS: reactive oxygen species, ATP: adenosine triphosphate, ADP: adenosine diphosphate) (This figure was drawn using the Bio Render app).

The Warburg and PPP are coincidentally regulated by several rate-limiting steps. These rate-limiting steps remain excitable targets for cancer targeting. Several specific metabolic inhibitors could be used as specific suppressors of enzymatic activity [78]. The small molecule inhibitors that have been reported to target glucose metabolism pathways and that have in vivo potency for reducing tumor volume will be discussed in this section (Figure 6).

GLUT inhibitor STF-31 can inhibit glucose transport by blocking the GLUT1 receptor. This small molecule inhibitor shows efficiency in inhibiting tumor xenografts. STF-31 has an off-target effect on nicotinamide phosphoribosyl transferase, thus making it insufficient for clinical application [138]. Another GLUT inhibitor, Glutor, has an inhibitory function on pan-GLUT receptors. Studies showed that Glutor can reduce glucose uptake in cancer cells and reduce ATP production [139]. BAY-897 is another pan-GLUT receptor inhibitor, which showed positive efficacy against triple-negative breast cancer cells [140].

Hexokinase serves as the first enzymatic reaction in the glycolysis pathway. Cancer cells especially upregulate HK2 expression. Many studies showed that loss of HK2 decreased tumor growth in vivo [141,142]. Thus, HK2 serves as a favorable target for cancer therapy and a rate-limiting step for the glycolysis pathway. A specific inhibitor of HK2, 3BP, showed a promising effect against various cancer models but possessed cross-reactivity with other pyruvylated proteins [143,144].

Pyruvate kinase is another potent target for inhibiting glycolysis in cancer cells. Lower PKM2 activity slows down the glycolytic flux in the cancer cells and thus serves as a rate-limiting enzyme for this pathway [145]. Loss of PKM2 shows a reduction in lactate formation, tumor volume, and an increase in infiltration of immune cells. It must be noted that deletion of PKM2 is not always tumor-inhibitory. In the colon and pancreatic cancer models, PKM2 deletion indeed increased tumorigenesis [146,147]. Thus, targeting PKM2 needed to be studied further for a better targeting effect. Shikonin, a small molecule isolated from *Lithospermum erythrorhizon*, confers an anticancer effect by inhibiting PKM2 activity in multiple cancer types [148,149]. PKM2 inhibition is also accompanied by ROS production, which induces cell death [150].

Lactate dehydrogenase, LDHA, and LDHB are important targets for cancer therapy. LDHA is upregulated in many cancer types. Silencing of LDHA reduces tumor mass irrespective of cancer xenografts [151,152]. The efficacy of the small molecule inhibitors of LDHA showed less potency due to low in vivo pharmacokinetic exposure. For instance, GSK2837808A physically has no efficacy in vivo, but makes the cancer cells more susceptible to tumor-infiltrating lymphocyte killing [153]. GNE-140, another LDHA inhibitor, acts on the pyruvate pocket of the LDHA enzyme. GNE-140 treatment increases OXPHOS levels driven by the AMPK (adenosine monophosphate-activated protein kinase)–mTORC1 pathway [154]. Possibly, GNE-140 would be effective in combination with AMPK or mTORC1 inhibitors. Studies showed LDHA and LDHB both function redundantly in cancer, so targeting both would be more advantageous for therapeutic purposes [155], In melanoma, cas9-mediated deletion of both isoenzymes showed favorable effects on tumor mass shrinkage. The drawback of targeting LDHA is the induction of hemolysis, as erythrocytes rely more on the glycolysis pathway [156,157].

Pyruvate dehydrogenase mediates the conversion of pyruvate to acetyl-CoA. The glucose uptake in melanoma cells was liaised by physiologically hyper-active pyruvate dehydrogenase kinase. PDK inhibitor DCA treatment in melanoma reduces cellular proliferation in vitro [158]. Until now, the use of DCA is not suitable for cancer clinical study. It is uncertain if previously investigated dosage ranges will result in cytotoxic intra-tumoral DCA concentrations. However, it has multiple clinical trial experiences with various other diseases with the consequences of lactic acidosis. Many additional mono- or di-halogenated short-chain fatty acid derivatives have been developed to activate PDC by inhibiting PDKs and to bind to the pyruvate site. In rat cardiac mitochondria, 2-chloropropionate has potency comparable to DCA, but it is too toxic for therapeutic application [95].

Inhibition of the lactate transporter has shown a greater possibility of therapy with various redundant side effects, such as elevation of urinary lactate levels. AZD3965, an MCT1 blocker, is now in phase 1 trials for advanced cancer stages. This small molecule showed promising inhibitory effects on MCT1 and 4 in pre-clinical studies [159,160]. 

Drug repositioning (DR), or screening for anticancer therapy properties of regularly prescribed pharmaceuticals for non-malignant illnesses, has attracted a lot of interest because the safety and frequency of side effects of these treatments have previously been established. The potential anticancer role of metformin is widely studied, but the mode of action of metformin in cancer is misleading. Several cancer-bound studies indicate that (I) metformin decreases blood glucose levels by decreasing hepatic glucose production (also called gluconeogenesis); (II) metformin inhibits mitochondrial complex I activity; and (III) metformin activates AMPK and phosphorylates two isoforms of the acetyl-CoA carboxylase enzyme, thereby inhibiting fatty acid synthesis and leading to fatty acid oxidation. Thus, metformin-induced energy deprivation inhibits proliferation in cancer [161,162,163].

Activation of G6PD produces NADPH. NADPH, in turn, has a role in fatty acid and steroid synthesis, as well as the maintenance of lowered GSH levels for antioxidant function. The PPP enzyme G6PD is another fruitful target for cancer therapy, as it maintains the redox balance and PPP flux in the cells. Studies showed that G6PD deficiency reduced cancer incidence and mortality in patients [164,165]. The natural molecule polydatin showed efficacy in inhibiting G6PD, followed by cell cycle block and apoptosis induction in cancer cells. Polydatin also makes the cells susceptible to ROS-mediated damage, which is a hallmark of G6PD inhibition [166]. 

## 10. Glucose Metabolic Reprogramming and Therapy Resistance

Tumor cells proliferate and grow in the presence of glucose in the tumor microenvironment. Cancer cells using glucose for growth and survival mainly develop therapy resistance and recurrence. In cervical cancer, cisplatin resistance develops due to elevated lactate levels [167]. Further, AMPK mediates metabolic reprogramming in therapy-resistant cancer cells by promoting the Warburg pathway and mitochondrial biogenesis [168]. As stated earlier, several oncogenic transcription factors (MYC, SIX1, etc.) act as inducers of glucose uptake and are used through aerobic glycolysis and the PPP process to promote DNA repair and apoptosis resistance.

Metabolic reprogramming in cancer cells supports DNA repair and suppresses oxidative stress and the immune system in the tumor microenvironment [169,170,171]. Furthermore, Warburg and PPP benefit cancer cells by evading anti-apoptosis by augmenting the autophagy pathway [172,173,174]. These are the basis for developing cancer therapy resistance. Chemo and radiotherapy primarily persuade oxidative burden in the cancer cells and eventually do irreversible damage to the DNA repair machinery [175,176]. Recent studies show breast cancer and mesothelioma cells upregulate aldehyde dehydrogenase (ALDH) to make the nucleotide pools available for DNA repair [177]. G6PD from PPP produces ribose-5-phosphate to enhance nucleotide synthesis. Both glycolysis and PPP can limit ROS production by accumulating pyruvate flux and NADPH production, respectively [150]. TP53-induced glycolysis and apoptosis regulator (TIGAR) (p53-target gene) has domain similarity with fructose-2,6-bisphosphatase (FBPase-2), and transforms fructose-2,6-bisphosphate to fructose-6-bisphosphate, thus limiting the activity of PFK1 and diverting glycolytic flux towards PPP for nucleotide production and DNA damage repair [178]. Oncogene MYCN can regulate G6PD through p53 inactivation and facilitate nucleotide synthesis and DNA repair in MYCN-amplified neuroblastoma [179]. DNA damage repair mediated by glucose metabolic reprogramming is a complex operation that involves the activation of multiple oncogenes, as well as the activation or inactivation of signaling pathways.

Induction of autophagy is another mechanism of cell survival in “chemotherapeutic” stressed conditions. Glucose deprivation therapy or 2-deoxy-D-glucose (2DG) treatment can block the glycolytic flux, but alternatively, it activates AMPK signaling-mediated autophagy [180]. Activated autophagy blocks apoptosis in cancer cells, and cells deplete intracellular energy reserves such as glycogen and proteins for survival. Autophagy upregulation driven by metabolic dysfunction may contribute to a common mechanism of resistance to chemotherapy and radiation by reducing apoptosis and acting as a pro-survival mechanism. Although autophagy induced by glucose dysfunction acts as a pro-survival mechanism, it remains controversial, as the autophagy inducer rapamycin shows treatment synergy with the metabolic inhibitor ponatinib against multiple myeloma [181].

The tumor microenvironment is crucial for tumor cells; they adapt to the available conditions and resources. For example, in hypoxia, HIF-1α transcriptionally controls multiple glycolytic and PPP pathway gene expressions, which sustain cells’ metabolic viability in adverse conditions. Moreover, HIF-1α can trigger the PD-L1 expression by binding to the hypoxic response element at PD-L1 gene promoter [182,183]. Higher PD-L1 on the cancer cells mask the T cell immunity to act on them [184]. Regardless of the complexity of the tumor microenvironment and the positive involvement of factors such as AKT-mTOR-HIF-1α in modifying glucose metabolism, this topic is still vastly open to novel research for a complete understanding.

## 11. Metabolic Alterations in Cancer Cells at Low Glycolytic State

Several anti-Warburg therapies are attracting attention in the research. Histone deacetylase (HDAC) inhibitors are capable of downmodulating the super-enhancer regions of several glycolytic enzymes, including HK2, GPI, glyceraldehyde-3-phosphate dehydrogenase (GAPDH), etc., but inhibition of the glycolytic pathway by HDAC inhibitors leads to the activation of alternative pathways to maintain the cellular energy homeostasis [11,185]. Certain other glycolytic targeting drugs, such as 2DG and lonidamine, have not been effective in clinical trials. In this section, we will briefly describe the alternative pathways that become dominant in Warburg’s suppressed condition.

In tumors, mitochondria perform a variety of critical activities, such as the dynamic regulation of all malignant cellular processes via a metabolic rewiring technique. Cancer cells use mitochondria as metabolic machinery to meet increased bioenergetic necessities through ATP generation, as well as increased anabolic requirements and the biosynthesis of nucleotides, lipids, and proteins. Furthermore, it has been established that glycolytic neoplastic cells preserve OXPHOS under glycolysis-inhibited circumstances [186]. The cancer cells use alternative carbon sources, such as glutamine, serine/glycine, and fatty acids, to feed the TCA cycle [27]. It has been demonstrated that the melanocyte-specific transcription factor (MITF) regulates the PGC-1 gene, resulting in higher PGC-1-dependent mitochondrial respiration. Maintaining an increased OXPHOS rate promotes tumor development and dissemination. Several kinds of cancer have been found in recent investigations to develop a greater OXPHOS dependency and an increase in aggressiveness [187]. Therefore, to maintain a constant energy source, cancer cells shift to the alternative pathway for energy homeostasis. By keeping the OXPHOS rate high, cancer cells promote tumor development and metastasis. From a therapeutic point of view, inhibition of OXPHOS along with Warburg inhibition is gaining attention. The carbonyl cyanide p-(tri-fluromethoxy) phenyl-hydrazone (FCCP), an inhibitor or uncoupler of OXPHOS, has been used to induce cell death. FCCP is also able to depolarize mitochondrial membrane potential [188]. The F0F1-ATP synthase activity blocker s-Gboxin also serves as an OXPHOS inhibitor and induces apoptosis in various cancers [189]. FAO produces acetyl-CoA units from fatty acids. Cancer cells with fast growth rates rely on the FAO pathway linked with lower glucose availability [81,127]. It has been noted that activation of the FAO pathway can actually elevate mitochondrial membrane potential and induce paclitaxel resistance in TNBC cells. Targeting the FAO component ASCL4 showed promising effects on tumor reduction in vitro and in vivo [190]. A recent finding showed that induced myeloid leukemia cell differentiation protein (MCL-1)-driven cancer cells are sensitive to FAO inhibition, and genetic deletion of MCL-1 induces global downregulation of the FAO pathway [191]. Therefore, the FAO pathway is very active in Warburg-inhibited cells and is responsible for apoptosis resistance. Researchers previously used clinically approved trimetazidine to counteract the FAO pathway. It decreases long-chain fatty acid oxidation by inhibiting 3-ketoacyl-CoA thiolase [192] and induces cell death in various cancers [193,194]. Alternatively, many amino acids activate mTOR in the form of the mTORC1 complex. mTORC1 regulates protein translation by activating the S6K kinase and inhibiting 4EBP1 to activate the protein synthesis complex [195]. 

## 12. Therapeutic Strategies

Targeting metabolism aims to inhibit glucose consumption to decrease the amount of ATP, reducing amino acids and nucleotide synthesis in the cancer cells. Treatment with the HK2 inhibitor 3-bromopyruvate reduces the aerobic glycolysis flux in cancer cells and destabilizes the redox state of the cancer cells [196]. The PFKFB3 glycolysis inhibitor, 3PO, can decrease glycolysis in nintedanib- and sunitinib-resistant tumor cells by causing cell-cycle arrest and death [197]. 

Glycolytic-inhibited cells are prone to OXPHOS addiction [198]. Metabolically altered cells are sensitive to OXPHOS inhibitors. The electron transport chain inhibitors could be used in combination to control OXPHOS levels in the cells. This could dismantle the energy balance of the cells. Metformin targets the electron transport complex and reduces the aggressiveness of the tumor. On the other hand, mTORC1 inhibitors are in the clinic as anticancer drugs. Rapamycin targets mTORC1 and, in turn, inhibits PGC1α activity to reduce FAO and OXPHOS pathways in cancer cells. By enhancing ROS generation, newly designed nanoparticles having metals (arsenic, iron oxide, and manganese) can lower mitochondrial function and OXPHOS, which efficiently trigger cancer cell death. Therefore, inhibiting OXPHOS may ensure anticancer medication effectiveness and reverse treatment resistance. Therapeutic targeting of electron transport chain components has been shown to enhance the anticancer efficacy of alisertib and cause severe energy drops in ATP-dependent mitotic cells. In vivo, tumor development is also reduced when metformin and alisertib are used together [199].

The metabolic inhibitors can reach their best biological activity only when they are paired with specific pathway inhibitors, cellular immune agonists, and agonists or inhibitors connected with alternative metabolic pathways. Furthermore, most metabolic inhibitors lack selectivity, meaning they cannot target tumor cells without also affecting normal cells. As a result, metabolic inhibitor research has strong development possibilities. 

## 13. Future Directions

The enzymes in glucose metabolism are well studied as cancer targets. Many small-molecule inhibitors of glucose metabolism are either in the preclinical or clinical stages. However, susceptibility to specific inhibitors as single treatments or in combination with chemotherapy, radiotherapy, targeted therapy (such as kinase inhibitors), and/or immunotherapy remains unknown. Several clinical studies are now underway that consider various metabolic inhibitors combined with FDA-approved chemo- or kinase-inhibitor molecules (Table 1). For instance, saxagliptin and metformin, which control type 2 diabetes through AMPK activation and acetyl-CoA inhibition, are now under clinical trials as a therapy for PCOS women with impaired glucose homeostasis [200]. Likewise, a kinase inhibitor, selumetinib, used for treating neurofibromatosis type 1, in combination with the mTORC1 blocker sirolimus, was under phase 2 trial [201]. Paclitaxel, a chemotherapy drug, is now being used in combination with telaglenastat, which is an investigational, first-in-class, selective, oral glutaminase inhibitor (study number NCT03057600). Combinations with dabrafenib (BRAF inhibitor), trametinib (MEK kinase inhibitor), and pembrolizumab (humanized antibody for cancer immunotherapy) are now in use with rosuvastatin (HMG-CoA reductase inhibitor (statins)) or BP-101 (polyamine metabolic inhibitor) against multiple cancers (Table 1). The future avenue of research could focus on identifying the metabolic targets of interest through unbiased CRISPR-Cas9 synthetic lethality screening of metabolic genes that can possess an antitumor response, particularly in vivo. These findings may further translate to the clinical phase for understanding the efficacy and toxicity to provide better support for cancer management.

## 14. Concluding Remarks

Metabolic plasticity is key for cancer cell growth and metastasis. Most cancer cells rely on aerobic glycolysis, or the Warburg process, for fast energy production. PPP is coupled with the glycolysis process to work on nucleotide synthesis. Targeted drugs or molecules for PPP do not exist in the clinic, but targeted inhibitors for Warburg pathways are now available. Unfortunately, the inhibition of glycolysis can reactivate alternative pathways that are recessive in normal conditions. Presently, novel strategies are being adopted to target multiple metabolic pathways to inhibit cancer growth and recurrence. As we discussed, targeting OXPHOS and the Warburg pathway together would be a practical therapeutic strategy to inhibit cancer aggressiveness.

## Figures and Tables

**Table 1:** List of clinical trials of metabolic inhibitors in cancer patients. The list of studies was obtained from https://clinicaltrials.gov (accessed on 15 August 2023). The therapy was either single-dose or in combination with other anticancer drugs.

NCT Number	Study	Drug	Phase
NCT02022007	PCOS Women with Impaired Glucose Homeostasis	Metformin XR, Saxagliptin	3
NCT03433183	Malignant Peripheral Nerve Sheath Tumors	Selumetinib, Sirolimus	2
NCT03057600	Advanced Triple Negative Breast Cancer (TNBC)	Paclitaxel, Telaglenestat (CB-839)	2
NCT02858921	BRAF Mutant Stage III Melanoma	Dabrafenib, Trametinib, Pembrolizumab	2
NCT04776889	Castrated Egyptian Prostate Cancer Patients	Rosuvastatin and surgical castration	4
NCT03428217	Metastatic Renal Cell Carcinoma	CB-839, Cabozantinib	2
NCT05254171	Pancreatic Cancer	SBP-101, Nab-paclitaxel, Gemcitabine	2/3
NCT03965845	Solid Tumors	CB-839, Palbociclib	1/2
NCT02771626	Melanoma, Clear Cell Renal Cell Carcinoma (ccRCC), and Non-Small Cell Lung Cancer (NSCLC)	CB-839, Nivolumab	1/2
NCT00859495	Pleural Mesothelioma	Doxorubicin, Cisplatin, Pemetrexed and Radiotherapy	2
NCT03163667	Renal Cell Carcinoma (RCC)	CB-839, Everolimus	2
NCT04207086	Stage III Melanoma	Pembrolizumab, Lenvatinib	2
NCT02903914	Advanced/Metastatic Solid Tumors	INCB001158, Pembrolizumab	1/2
NCT00360828	Recurrent Anaplastic Astrocytomas, Mixed Malignant Gliomas, and Oligodendrogliomas	Irinotecan Hydrochloride	2
NCT03449901	Soft Tissue Sarcoma, Osteosarcoma, Ewing’s Sarcoma, and Small Cell Lung Cancer	pegylated arginine deiminase, Gemcitabine, Docetaxel	2
NCT05796570	AML, MDS, and Related Myeloid Malignancies	Decitabine, Filgrastim	2
NCT03875313	Solid Tumors	CB-839, Talazoparib	1/2
NCT00634270	Plexiform Neurofibromas	Sirolimus	2
